# Gene network reveals *LASP1, TUBA1C*, and *S100A6* are likely playing regulatory roles in multiple sclerosis

**DOI:** 10.3389/fneur.2023.1090631

**Published:** 2023-03-09

**Authors:** Nafiseh Karimi, Majid Motovali-Bashi, Mostafa Ghaderi-Zefrehei

**Affiliations:** ^1^Department of Cell and Molecular Biology and Microbiology, Faculty of Biological Science and Technology, University of Isfahan, Isfahan, Iran; ^2^Department of Animal Genetics, Yasouj University, Yasuj, Iran

**Keywords:** multiple sclerosis (MS), Bayesian network, transcriptome, Cytoscape, qPCR

## Abstract

**Introduction:**

Multiple sclerosis (MS), a non-contagious and chronic disease of the central nervous system, is an unpredictable and indirectly inherited disease affecting different people in different ways. Using Omics platforms genomics, transcriptomics, proteomics, epigenomics, interactomics, and metabolomics database, it is now possible to construct sound systems biology models to extract full knowledge of the MS and recognize the pathway to uncover the personalized therapeutic tools.

**Methods:**

In this study, we used several Bayesian Networks in order to find the transcriptional gene regulation networks that drive MS disease. We used a set of BN algorithms using the R add-on package bnlearn. The BN results underwent further downstream analysis and were validated using a wide range of Cytoscape algorithms, web based computational tools and qPCR amplification of blood samples from 56 MS patients and 44 healthy controls. The results were semantically integrated to improve understanding of the complex molecular architecture underlying MS, distinguishing distinct metabolic pathways and providing a valuable foundation for the discovery of involved genes and possibly new treatments.

**Results:**

Results show that the *LASP1, TUBA1C*, and *S100A6* genes were most likely playing a biological role in MS development. Results from qPCR showed a significant increase (*P* < 0.05) in *LASP1* and *S100A6* gene expression levels in MS patients compared to that in controls. However, a significant down regulation of *TUBA1C* gene was observed in the same comparison.

**Conclusion:**

This study provides potential diagnostic and therapeutic biomarkers for enhanced understanding of gene regulation underlying MS.

## 1. Introduction

Multiple sclerosis (MS) is a multifocal inflammatory autoimmune disease (1). Even though MS is usually considered a white matter disease, but several studies have demonstrated the involvement of gray matter impairment in conjunction with cortical and deep (2–6) leading to progressive neuronal damage in genetically sensitive hosts (1). MS is a complex multicomponent demyelinating disease and its pathophysiology consist of redox, autoimmune, vascular, and neurodegenerative systems, to name a few. The clear-cut mechanisms of MS triggering, its development, and progression are still obscure. In MS, impairing of the myelin sheath of neural axons in the Central Nervous System (CNS) is observed (7, 8). MS shows a long range of symptoms e.g., from pathological processes to severe physical disabling. Gender preferences, genetical factors, and geographical differences have been reported for people suffering from MS. Over outburst of MS, monocytes, which are a preserved subset of white blood cells, are activated by interferon-β (IFN-β) (7). MS study is quite vivid, using Omics data, many authors have used gene networks to get some insight into molecular mechanisms of MS (9–19). The integration of information gleaned from a variety of resources encompassing transcriptomics, genomics, proteomics and patient clinical data could boost our understanding of the mechanism(s) underpinning the reason for this disease (20). In this regard, we can explore the signaling pathways involved in MS (21), apply logical networks to model signaling pathways in MS (22) and use networks to combine information on transcriptome-interactome data from MS studies (17). We can also apply theory of biochemical systems for improving therapeutic drugs in re-myelination (15), create molecular networks based on transcription factors and genes expressed in mononuclear cells in MS patients (23), and design reactive networks between distinct miRNA and target genes in T cells (23). This approach will help explain the molecular mechanisms of the MS disease (12). Supplementary Table 1 shows some examples of network-based studies used with different MS biological data. As one can see, Bayesian Network (BN) modeling paradigms have rarely been applied in this setting. BN uses probability theory to reason under uncertainty. BN as a graphical scheme (directed acyclic graph) consists of a qualitative part (structural model) and a quantitative part (local probability distributions), which allow for a different kind of probabilistic inference, and quantitatively measures even the smallest impact of a variable or set of variables on others. This sort of modeling is of great importance in transcriptomic studies, since it can reveal both qualitative and quantitative elements of learned gene networks. BN has previously been used in several transcriptomic studies (10, 24, 25).

Many existing categories of gene networks identify groups of related genes as gene sets, making experimental follow-up a formidable task. With BN, it is possible to determine whether a gene is a driving source of changes in its gene network or not, since both in-degree and out-degrees of connectivity of each gene can be readily verified. The more out-degree gene has, the higher likelihood of being a possible regulator one. This would be a crucial characteristic for example when looking for potential drug targets. However, it is likely that a specific transcription factor defining a particular cell type that drives pathology, may not have a large number of out-degrees while still being crucial. To this end, if a particular gene is expressed across different cell types, for example like *S100A6*, then it may be correlated with different genes, but this may be a spurious correlation. Therefore, to leave off possible artifacts, we should use extra source of information when interpreting the results. Today, MS research is increasingly data-driven—a trend that arguably shall continue at a much higher rate in times to come. To tackle these large amounts of heterogeneous data, and to derive insight into MS disease, many interdisciplinary scientists have started using a variety of computational tools. In this study, we aim to gain much insight into the regulatory transcriptional gene network underlying MS using systems biology approaches in the context of BN, that may yield mechanistically interpretable results.

## 2. Methods

### 2.1. Network analysis

In this study, the Gene Expression Omnibus (GEO) database (https://www.ncbi.nlm.nih.gov/geo/) was scanned using a combination of several simple key words, and resulting DNA microarray experiments related to MS, that fulfilled our criteria. In the end, based on our criteria for choosing a suitable GEO data set, the microarray series with accession number GSE17048 was downloaded from GEO using the GEO query package (26). This accession was seen to have the highest number of arrays per probe—a fact that would help minimize the rate of false positives while training the regulatory gene BN. The GSE17048 contained 56 blood samples from MS group [44 patients were in the RRMS phase (relapsing-remitting) and 12 patients were in the SPMS phase (progressive-secondary)]. The control population was 44 healthy people without any symptoms. The average age of the patients was 39.5 years old and the control group was 39.23 years old, and in terms of gender, the MS included 29 women and 15 men (higher prevalence of the disease in women) and 23 men and 21 women were studied in the control group. In order to remove noise from the data, probes with the highest variance were obtained and used as an input to train the gene regulatory BN using bnlearn, an R add-on package (27, 28). The following codes were used to filter the probes with highest variances: *qt* <*- quantile[t(data1); probs* = *c(0.0002,0.99)]; rows1* <*- apply[t(data1), function(x) any(x* < *qt | x* > *qt)]; data2* <*- t(data1)[, rows1]*. We obtained the best fitted BN model on our data using Bayesian information criterion (BIC) and its adjacency matrix, with the help of the *Cytoscape*-based *aMatReader* plugin, with the *Cytoscape* (29) environment used for further downstream scrutiny.

### 2.2. Downstream analysis

**Figure 1:**
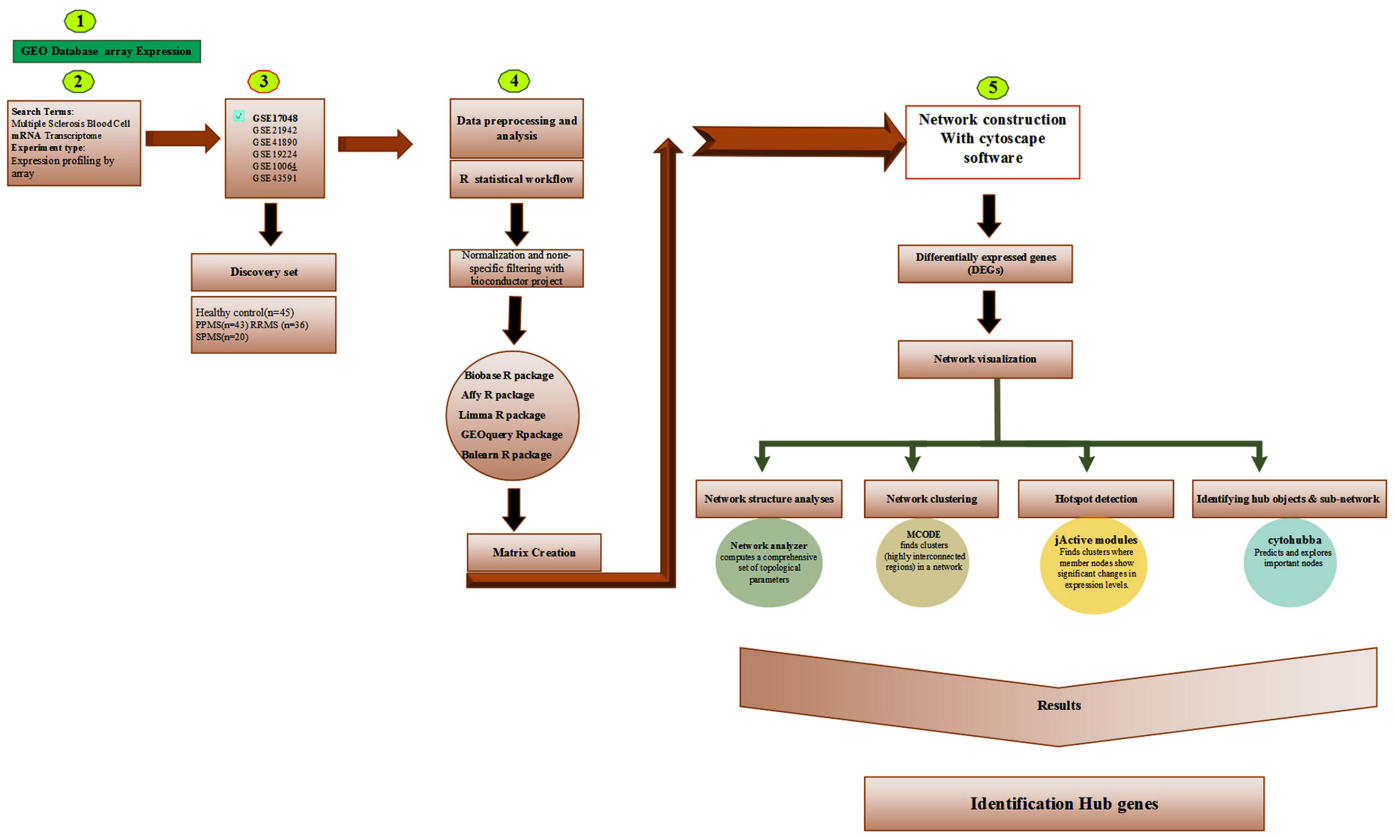
The analysis pipeline used in this study^1^^,^^2^^,^^3^^,^^4^.

This was accomplished with the following *Cytoscape* add-on packages. The *jActiveModules* were used to explore the concept of gene modules and find sub-networks (30); *MCODE* to identify putative complexes by finding regions of significant local density (31); *CytoHubba* to explore the protein-protein interaction (PPI) network of hub genes using eleven different methods (32). The *NetworkAnalyzer* was used to determine the hub genes, taking into account the degree of topological criteria (e.g., the number of nodes, edges, and connected components, along with the network diameter, radius, density, centralization, heterogeneity, clustering coefficient, the characteristic path length, the distributions of node degrees, neighborhood connectivity, average clustering coefficients, and shortest path lengths) (33). The *iRegulon* was used to detect targets / motifs/paths from a set of genes; and the *CyTargetLinker* to integrate regulatory reactions in network analysis. In addition, we used *Metascape* (34) to annotate the multiple gene lists in our study. Even though transcriptomic statistical analysis is generally based on probe level data, the probe names were converted to their corresponding gene names using g:Profiler (https://biit.cs.ut.ee/gprofiler/gost) to get better insight into the data. Results from the aforementioned software were combined. Figure 1 shows the analysis used in this study.

### 2.3. Validation of *LASP1, TUBA1C*, and *S100A6* genes using quantitative real-time PCR

#### 2.3.1. Ethics statement

Following the bioinformatics analysis, validation of significant differentially expressed genes (DEGs) (*LASP1, TUBA1C*, and *S100A6*) was carried out using QRT-PCR. A total of 100 whole blood samples (56 MS cases, mean age: 39.5 years and 44 controls, mean age: 39.5 years), obtained from MS Research Center and Al-Zahra Hospital in Isfahan (http://alzahra.mui.ac.ir) were used. All procedures were approved and carried out in accordance with Medical Research Ethics Committee of Iran under code IR.UI.REC.1399.076.

#### 2.3.2. RNA extraction

Total RNA was extracted from each sample according to the standard TRIzol protocol (Bio BASIC, Canada) according to manufacturer's instructions. RNA concentration and quality were determined using both Nanodrop (Thermo Scientific Tm Nano Drope One C model) and gel electrophoresis. The existence of two sharp bands representing 18S and 28S ribosomal RNA on a 1% (w/v) ethidium bromide stained agarose gel during electrophoresis through TAE buffer (40 mM Tris-acetate, 1 mM EDTA, pH 8.0) at 100 V for 30 min confirmed the quality of the extracted RNAs. Those RNA samples with a RNA integrity Number (RIN) < 1.8 were excluded from further analysis. For all the RNA work DEPC-Treated Water was used. High quality extracted total RNA was stored at −70°C until cDNA synthesis.

#### 2.3.3. cDNA synthesis

Initially, DNAse I (Fermentase Cat # ENO 521) treatment was used to remove genomic DNA before cDNA synthesis. Next, cDNA synthesis was carried out using a commercial kit provided by Yektatajhiz Company (Cat No.: YT4500) according to manufacturer's instructions. This involved keeping the samples on ice under sterile conditions at 70°C for 5 mins, 37°C for 60 mins, 70°C for 5 mins, and finally storing all synthesized cDNAs at −20°C.

#### 2.3.4. Quantitative real time PCR analysis

To enable the validation of our candidate genes (*LASP1, TUBA1C*, and *S100A6*), SYBR Green -based QRT-PCR was performed using a LightCycler^®^ 96 (BioRad, Germany). The sequence of all primers used are listed in the Table 1. These were designed using the PRIMER3 program (http://frodo.wi.mit.edu). QRT-PCR reactions were performed in duplicate and the values of average cycle threshold (Ct) were determined for each sample. The conditions of QRT-PCR amplification were: 1 cycle at 95°C for 2 min, 40 cycles at 95°C for 50 s, 60°C for 30 s. The *human beta-actin* gene (*ACTB_HUMAN*) was used as the internal control. Hence, all calculated concentrations are relative to the concentration of the standard, expressed in arbitrary units and the quantification cycle values were automatically calculated with Rotor-Gene software version 6.1.

**Table 1:** Primers designed for QRT-PCR.

**No**.	**Gene**	**Name**	**Seq.(5-3)**	**TM**
1	*H-TUBA1C*	F	TTCCACCCTGAGCAACTC	60
		R	AACCAAGAAGCCCTGAAG	
2	*H-S100A6*	F	AGCACACCCTGAGCAAGA	60
		R	TCACCTCCTGGTCCTTGT	
3	*H-LASP1*	F	GAGCAGCAGCCTCACCAC	64
		R	TACCGCTTCCCGCCAC	
4	*β-actin*	F	TGGAGGTACCACCATGTACC	60
		R	CACATCTGCTGGAAGGTGGA	

The results were analyzed using the 2^−ΔΔCt^ method (35). In this study, beta-actin gene (as a reference gene) and *S100A6, TUBA1C*, and *LASP1* genes [as target genes (TRG)] and CT data from real-time expression of *TUBA1C, LASP1*, and *S100A6* were statistically analyzed (*P* < 0.05) by REST 2009 software. After checking the normality of data, using the Kolmogorov Smirnov test and the unpaired *t-*test in GraphPad Prism 8 software, a significant difference in the expression levels of genes *LASP1, TUBA1C*, and *S100A6* was observed between patients and healthy individuals.

## 3. Results

The fundamental idea behind this analysis was to shed some light into gene-gene interactions underpinning MS disease with regard to cause and effect (36). In this study, we reused GSE17048 experiment data which contained the profiled mRNA expression for all known genes in whole blood from 144 health individuals, 99 with MS (43 PPMS, 36 RRMS, and 20 SPMS). As meta-data of GSE17048 shows in the Gene Expression Omnibus–NCBI, in the conducting the experimental design, whole blood mRNA expression was compared between different types of MS and age-matched healthy control. The nature of probability distribution induced by a gene regulatory BN will allow diverse probabilistic gene queries to be answered in linear time. This makes BN to be practically appealing. The results of comparison of the network structures determined from various algorithms, including Hill Climbing, Tabu Search, Max-Min Hill Climbing, and Restricted Maximize algorithms with different scoring functions, are shown in Table 2. Some key properties of BN are fundamental in judging estimated results.

**Table 2:** Estimation of structural Bayesian network parameters with different algorithms.

**Parameters**	**Score based algorithm**		**Hybrid algorithm**	
	**Hill climbing**	**Tabu search**	**Max-min hill climbing**	**Restricted maximize**
No. of Nodes	1,707	1,707	1,707	1,707
No. of Arcs	1,700	1,500	2,485	2,188
Undirected arcs	0	0	0	0
Directed arcs	1,700	1,500	2,485	2,188
Markov blanket	3.32	2.69	4.64	3.93
Neighborhood size	1.99	1.76	2.91	2.56
Branching factor	1	0.88	1.46	1.28
No. of tests	4,354,565	413,365	9,721,786	7,228,057
loglik-g	−1,308,146	−1,308,146	−1,318,282	−1,313,436
AIC-g	−1,312,560	−1,312,560	−1,323,884	−1,319,335
BIC-g	−1,319,115	−1,319,115	−1,332,202	−1,328,094

loglik-g, The multivariate Gaussian log-likelihood (loglik-g) score; AIC-g, Akaike Information Criterion score; BIC-g, Bayesian Information Criterion score.

**Figure 2:**
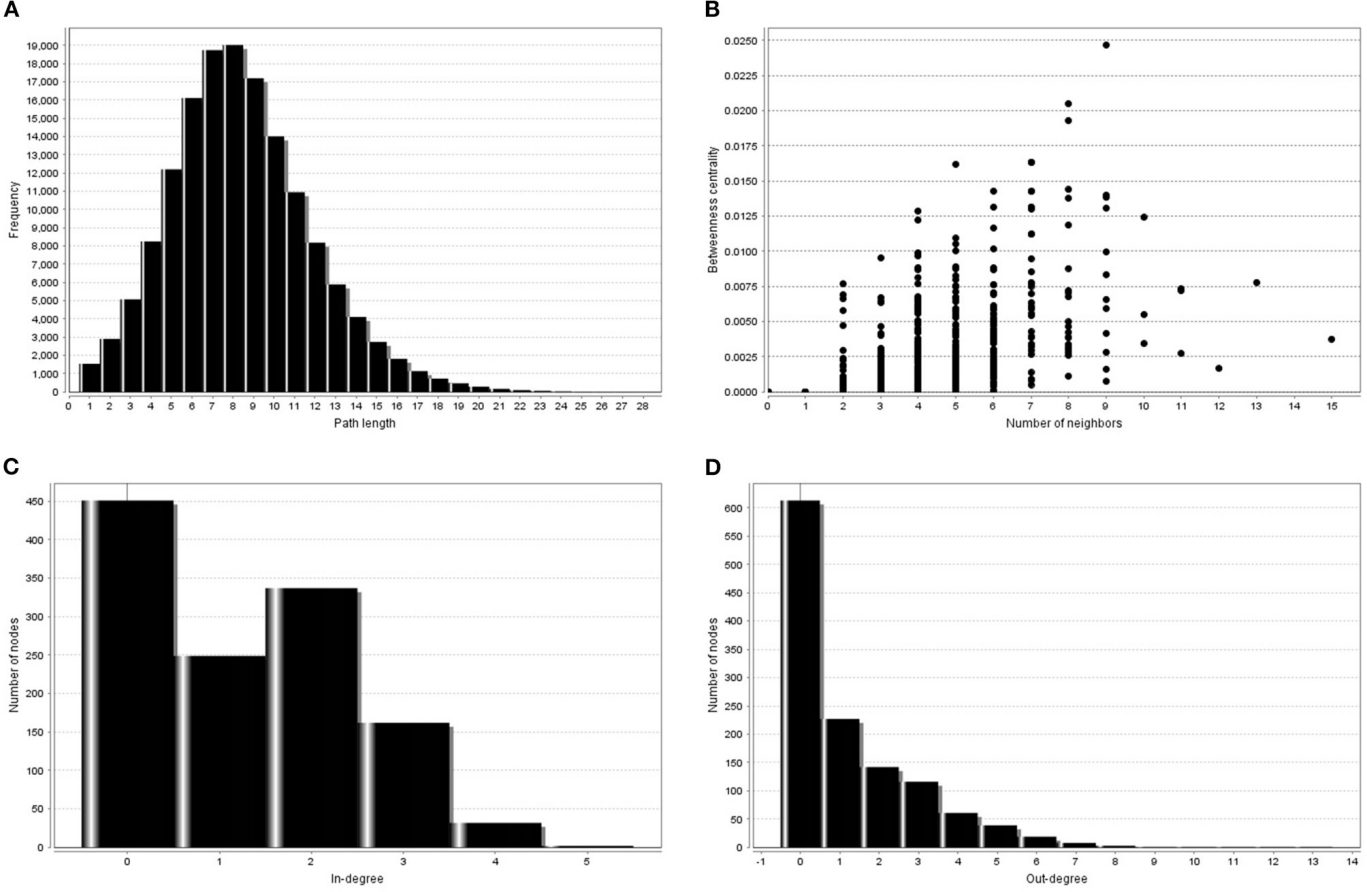
Some topological measures of trained gene regulatory BN visualized by *NetworkAnalyzer*. **(A)** Shortest path length distribution. The path length is the number of edges along the path. The distance d_ij_ between a pair of different nodes i and j is the length of the shortest path connection. **(B)** Between-ness centrality. The Between-ness centrality of a node reflects the amount of control that this node exerts over the interaction of other nodes in the network. **(C)** Distribution of in-degree gene connectivity measure. **(D)** Distribution of out-degree gene connectivity measure.

Figure 2 shows some properties of trained BN gene networks.

**Figure 3:**
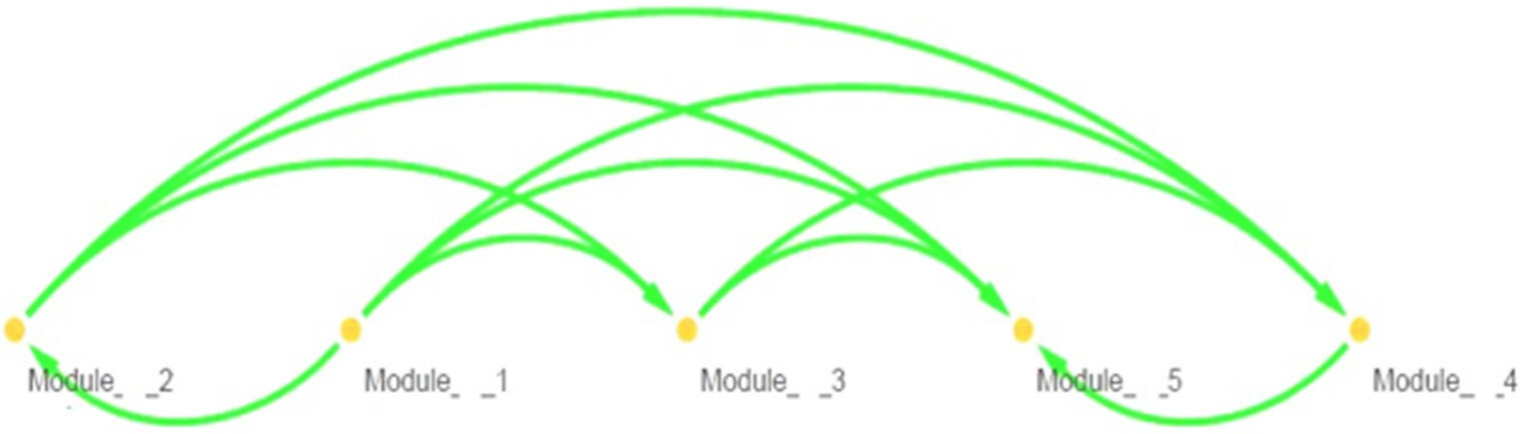
Extracted molecular modules using jActiveModules.

Topological parameters can characterize the location of genes in a gene network (37). Using *NetworkAnalyzer*, the following network topological parameters were calculated in our data. This was based on clustering coefficient (0.003), number of nodes (1,707), connected components (857), network diameter (26), network radius (1), shortest paths (192,463), characteristics path (8.313), the average number of neighbors (1.992), network density (0.0), isolated nodes (854), number of self-loops (0), multi-edge node pairs (0), and analysis times (1.467). The nature of probability distribution induced by a gene regulatory BN allowed diverse probabilistic gene queries to be answered in linear time. However, many structural BN parameters may be important. One of the key parameters (shown in Table 2) is the branching factor. This parameter plays a significant role in development of the gene network. Each Node (gene) will have its own branching measure, which will determine the out degree of that gene. If the branching factor value is not uniform in the network, an average branching factor can generally be calculated. This value turned out to be different depending on the type of algorithm used. Max-Min Hill Climbing returned a higher average than Restricted Maximize. In terms of system level understanding of research, the higher the branching factor, the more frequently gene regulators can be identified in the network. Biological networks have a modular architecture (38). *MCODE* can find connected and dense areas of the gene network based on network topology measures. In our analysis, 12 different modules were detected using *MCODE*, among which, 7 modules had 3 nodes; 3 edges with different interaction modes; 3 modules had 6 nodes and 7 edges; 1 module 15 nodes and 17 edges and finally 1 module had 6 nodes and 6 edges (Supplementary Figure 1). The active subnetworks were obtained using *jActiveModules*. The *jActiveModules* comprised 5 modules, where ILLMN_1742167 (*TUBA1C*), ILLMN_1665909 (*LASP1)*, and ILLMN_1713636 (*S100A6*) were seen to be enriched modules (Figure 3). The number of modules detected by this method was different than those identified with the *MCODE* based method.

**Figure 4:**
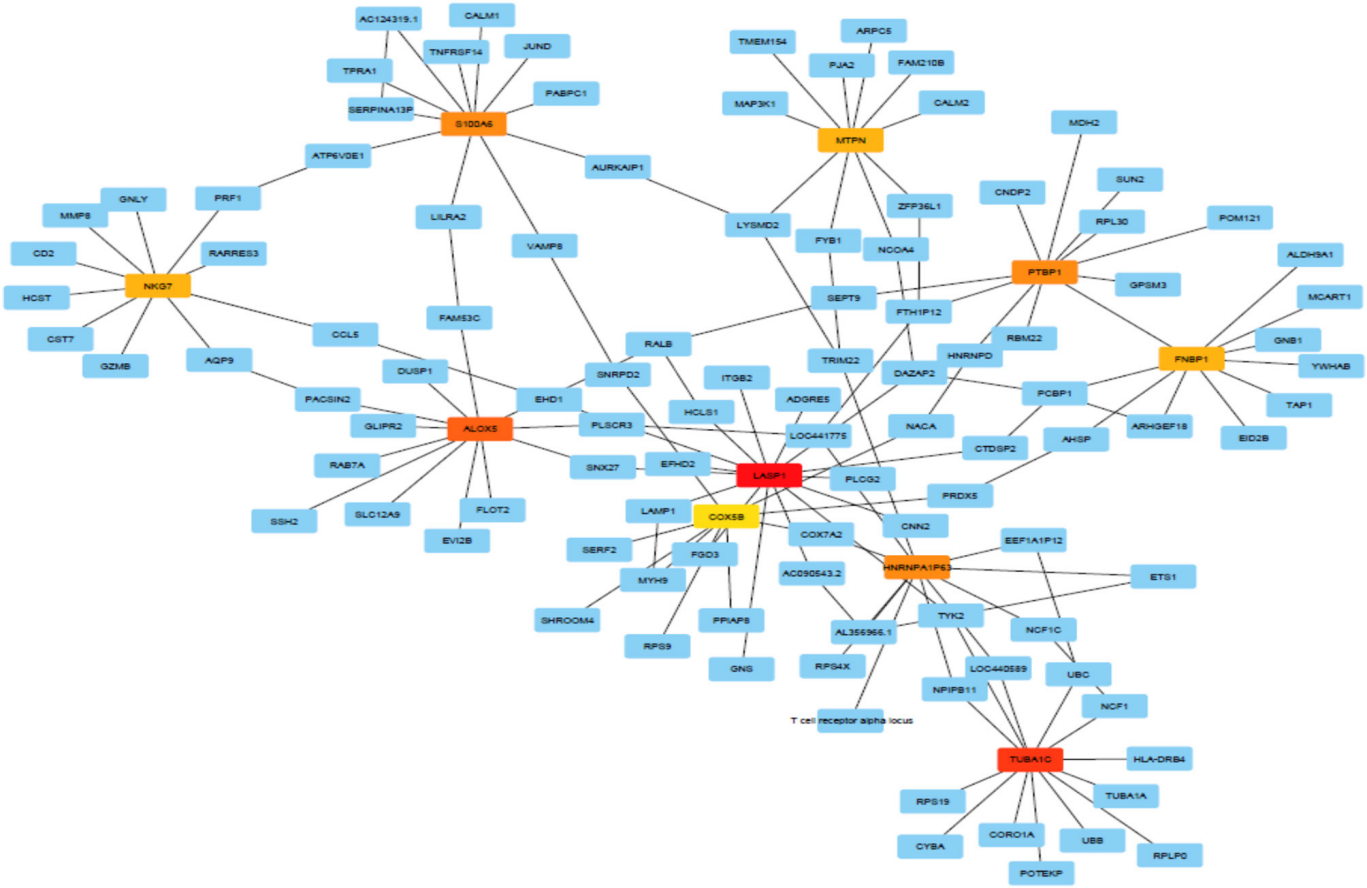
The MCC method captures essential genes in the top ranked list.

Figure 3 shows predicted modules in different modes of interaction. Module-level analysis explores the organization of biological systems and reconstructs module networks [A network module is a group of nodes (hub genes) that work together. Node or Vertex is a connection point or a branch point or an end point in a connection. And the path that connects the nodes to each other is called edge]. Figure 3 shows a module-level view of our gene regulatory BN network that denotes a high-level representation of the regulatory machinery of the MS gene network topology. Dense module searching of two MS Genome-Wide Association Study (GWAS) datasets identified several genes (*GRB2, HDAC1, IL2RA, JAK2, KEAP1, MAPK1, RELA*, and *STAT3*). These genes were enriched for glial cell differentiation (14). *CytoHubba* provides a user-friendly interface for discovering important nodes in biological networks (32). *CytoHubba* considers the shortest path between groups of nodes. Among the 11 proposed algorithms, MCC fitted better than the others. In Figure 4 and Table 3, we present the top 10 identified probes. Many of the genes, such as *TUBA1C, LASP1*, and *S100A6* shown in Figure 4, are close to the hub genes and were actually identified as hub genes by other algorithms such as *CyTargetLinker. The iRegulon* software then allowed us to identify regulons using motif discovery in a set of regulated genes. Identified transcription factors affecting the hub genes are listed in Supplementary Table 2 and Supplementary Figure 2 and their common factors identified are given in Table 4. The most significant, the STAT5A protein, mediates the responses of many cell ligands, such as IL2, IL3 and different growth hormones. In this study, the gene identifiers were uploaded to Metascape and used in conjunction with KEGG pathways, GO biological processes, Reactome gene complexes, canonical and CORUM pathways (39). The results of the enrichment analysis, including descriptions, function, ontology, expression, etc. are shown in Table 5, Supplementary Table 3, and Supplementary Figure 3.

**Table 3:** The 10 top genes/probes identified by the MCC method.

**Probe ID**	**Transcript ID**	**Gene Name**
ILMN_2180682	ENSG00000105887	“MTPN”, myotrophin
ILMN_1797342	ENSG00000187239	“FNBP1”, formin binding protein 1
ILMN_1713636	ENSG00000197956	“S100A6”, S100 calcium binding protein A6
ILMN_1663512	ENSG00000135940	“COX5B”, cytochrome c oxidase subunit 5B
ILMN_2333319	ENSG00000011304	“PTBP1”, polypyrimidine tract binding protein 1
ILMN_1792150	ENSG00000012779”	“ALOX5”, arachidonate 5-lipoxygenase
ILMN_1682993	ENSG00000105374	“NKG7”, natural killer cell granule protein 7
ILMN_1665909	ENSG00000002834	“*LASP1*”, *LIM* and *SH3* protein 1
ILMN_1742167	ENSG00000167553	“*TUBA1C*”, tubulin alpha 1c
ILMN_1691611	ENSG00000227453	“*HNRNPA1P63*”, heterogeneous nuclear ribonucleoprotein A1 pseudogene 63

**Table 4:** The top 10 transcription factors (TFs) estimated to affect hub genes.

**Transcription factors (TF)**	NES^*^	AUC^**^	**Target genes**
*STAT5A*	3.017	0.043	*LASP1, TUBA1C*
*NFATC1*	3.12	0.044	
*MTA3*	3.193	0.044	
*NFKB1*	4.005	0.037	
*ZNF362*	3.381	0.026	
*SPI1*	6.961	0.037	*LASP1, S100A6*
*GABPB1*	4.865	0.03	
*DLX1*	3.354	0.046	*TUBAC, S100A6*
*YY1*	6.592	0.036	*LASP1, TUBA1C, S100A6*
*NFATC3*	5.383	0.032	

* Normalized enrichment score (NES).

** Area under the cumulative recover.

**Table 5:** Metascape results *LASP1, TUBA1C*, and *S100A6* genes.

**Input ID**	**Gene ID**	**Tax ID**	**Gene symbol**	**Description**	**Biological process (GO)**	**Subcellular location (Protein atlas)**
*LASP1*	3927	H. sapiens	*LASP1*	LIM and SH3 protein 1	GO: 0034220 ion transmembrane transport; GO: 0009967 positive regulation of signal transduction; GO: 0023056 positive regulation of signaling	Cytosol; Plasma membrane (Supported) Focal adhesion sites (Approved)
*TUBA1C*	84790	H. sapiens	*TUBA1C*	tubulin alpha 1c	GO: 0030705 cytoskeleton-dependent intracellular transport; GO: 0000226 microtubule cytoskeleton organization; GO: 0051301 cell division	Microtubules (Supported)
*S100A6*	6277	H. sapiens	*S100A6*	S100 calcium binding protein A6	GO: 0048146 positive regulation of fibroblast proliferation; GO: 0048145 regulation of fibroblast proliferation; GO: 0007409 axonogenesis	Cytosol; Plasma membrane (Enhanced)

Genes were ranked from top to bottom based on degree, closeness and betweenness [higher degree (hub), higher betweenness (throat) and higher closeness centrality (shortest distance with other genes in the network)]. In terms of these parameters, three genes (*LASP1, TUBA1C*, and *S100A6*) showed a significant correlation with MS disease. These were thus identified as hub genes (Supplementary Table 4). In this study, probes ILLMN_1665909, ILLMN_1742167, and ILLMN_1713636 had high degrees of 15, 13, and 11, respectively and were identified as hub probes. In total of 850 probes had zero input edges, 200 probes had 1 in-degree. ILLMN_1665909, with the highest out-degree (13 out-degree) and 2 in-degree (mapped to human *LASP1*) plays an important role in regulating activity. Its encoded cytoplasmic protein binds focal adhesion proteins and plays a role in cell signaling, migration, and proliferation. ILLMN_1742167, with 12 out-degree and 1 in-degree mapped to the *human tubulin* gene (*TUBA1C*), and ILLMN_1713636 with 9 out-degree and 2 in-degree mapped to the *S100A6* gene (Figure 2).

### 3.1. Real-time reverse transcription polymerase chain reaction

**Figure 5:**
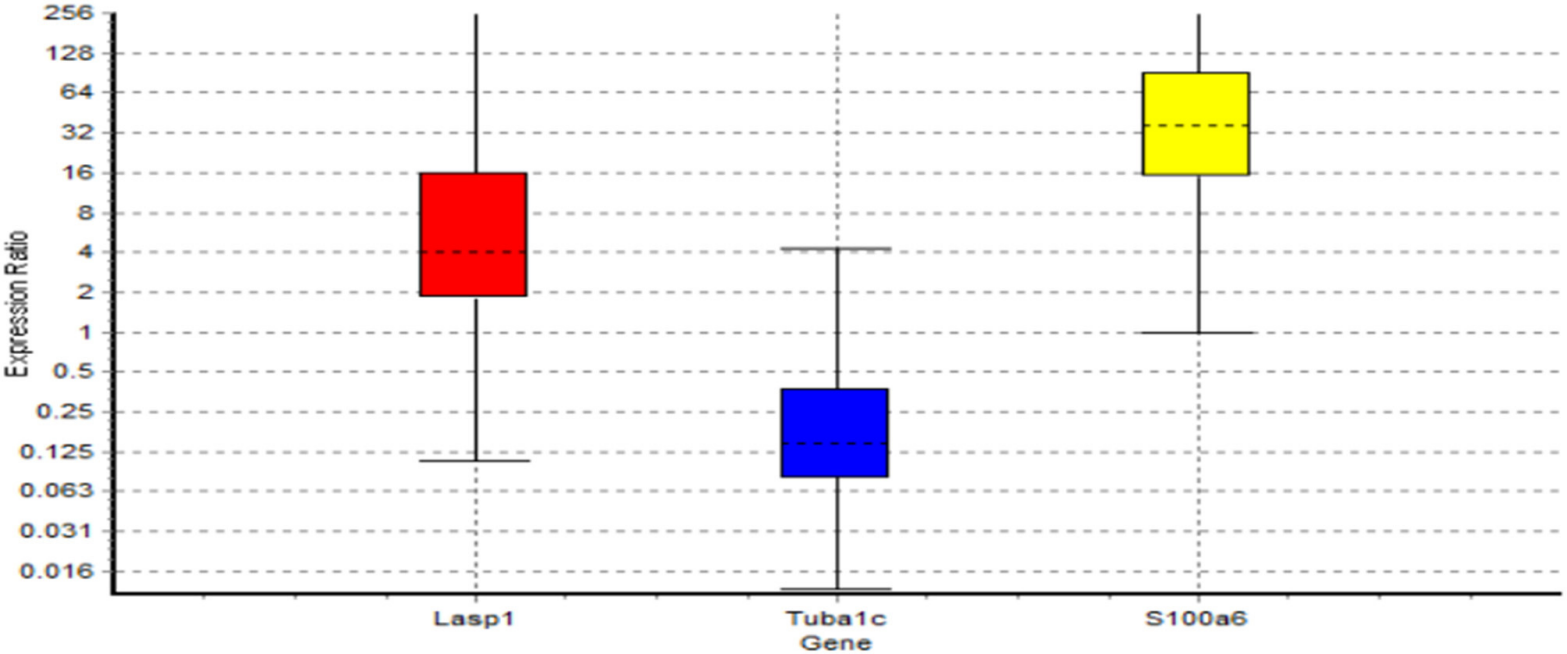
Boxes represent the interquartile range, or the middle 50% of observations. The dotted line represents the median gene expression. Whiskers represent the minimum and maximum observations.

As given in the Material and Methods section, we used RT-PCR to validate the results of Bayesian gene network. RT-PCR, that actually reflects product accumulation, is a routine lab-based method to validate array based transcriptomic results. In this study, the *LASP1, TUBA1C*, and *S100A6* genes turned out to be playing regulatory roles in MS. In validating aforementioned genes, using RT-PCR experiment, it was indicated that the patterns of relative gene expression for these genes (*LASP1, TUBA1C*, and *S100A6*) were significant between MS cases and controls (*P* < 0.05). The calculations based on the formula –ΔΔct shown the amount of mRNA transcripts of *LASP1* and *S100A6* genes, increased (5.491 and 36.556 times respectively) in patients though a decrease (0.166 times) in *TUBA1C* gene expression was seen in MS patients (*P* < 0.05) (Table 6 and Figure 5).

**Table 6:** REST software data compared *LASP1, TUBA1c*, and *S100A6* genes in MS patient and control groups.

**Gene**	**Type**	**Reaction efficiency**	**Expression**	**Std. error**	**95% C.I**.	**P(H1)**	**Result**
*ACTB*	REF	1.0	1.000				
*LASP1*	TRG	1.0	5.491	1.183–32.843	0.335–266.871	< 0.001	UP
*TUBA1C*	TRG	1.0	0.166	0.058–0.523	0.027–1.417	< 0.001	DOWN
*S100A6*	TRG	1.0	36.556	9.630–140.562	2.367–416.452	< 0.001	UP

P(H1), Probability of alternate hypothesis that difference between sample and control groups is due only to chance; TRG, Target; REF, Reference; Interpretation: LASP1 is UP-regulated in sample group (in comparison to control group) by a mean factor of 5.491 (S.E. range is 1.183–32.843); LASP1 sample group is different to control group. P(H1) = 0.000. TUBA1c is DOWN-regulated in sample group (in comparison to control group) by a mean factor of 0.166 (S.E. range is 0.058–0.523). TUBA1c sample group is different to control group. P(H1) = 0.000. S100a6 is UP-regulated in sample group (in comparison to control group) by a mean factor of 36.556 (S.E. range is 9.630–140.562). S100A6 sample group is different to control group. P(H1) = 0.000.

**Figure 6:**
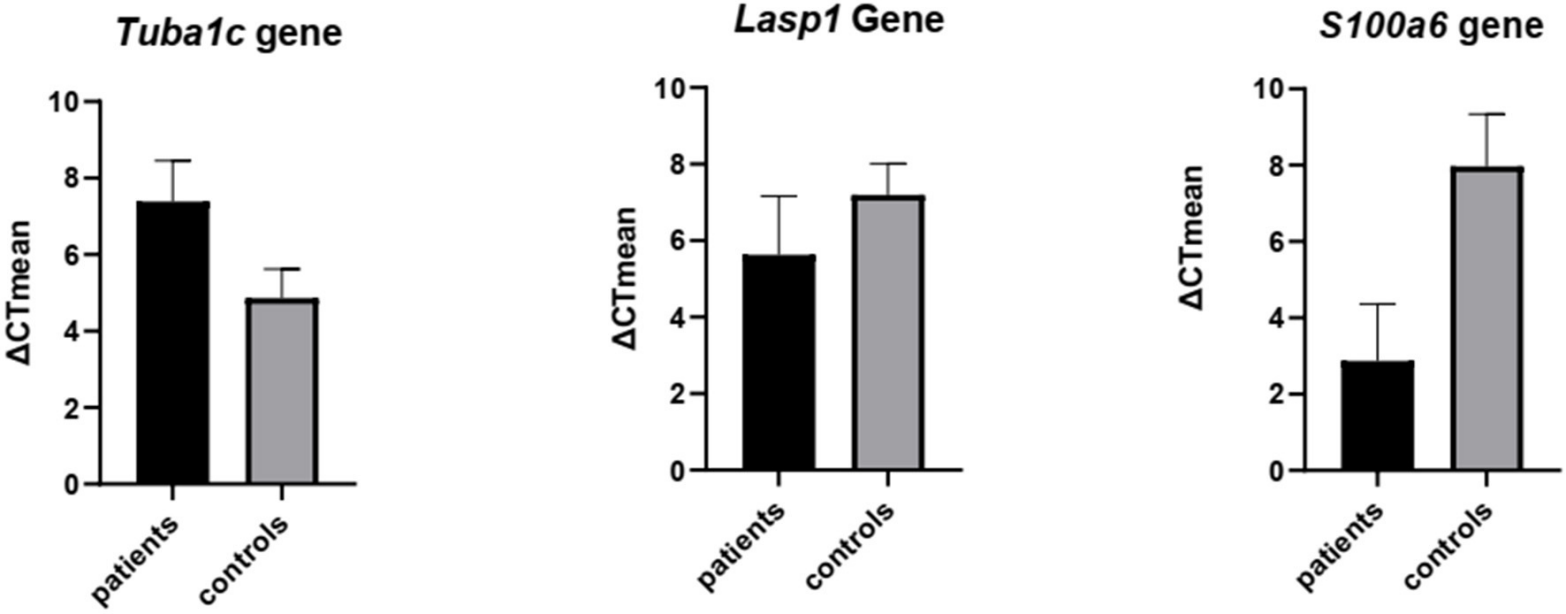
Validation of the expression of *TUBA1C, LASP1*, and *S100A6* genes by real-time PCR. Unpaired *t-*test was used to detect differences in gene expression between 2 groups patients & controls using the Graph Pad Prism 8 software. Significance: *P*-value TUBA1C < 0.0001, *P*-value S100A6 < 0.0001, *P*-value LASP1 < 0.003.

The results after studying the normality of the distribution of variables using the one sample Kolmogorov–Smirnov test and unpaired *t-*test in GraphPad Prism 8 software show a significant difference in expression levels of *LASP1, TUBA1C*, and *S100A6* genes between patients and healthy controls. *P-*values were: TUBA1C < 0.0001, S100A6 < 0.0001, LASP1 < 0.003. Mean expression of *TUBA1C, LASP1*, and *S100A6* genes in patient samples was 7.4, 5.6, and 2.9 respectively and 4.9, 8.1, and 8.0, respectively in healthy individuals. Results from statistical analysis also showed a decrease in *TUBA1C* gene expression and an increase in *LASP1* and *S100A6* gene expression in MS patients compared to the control group (Figure 6).

## 4. Discussion

At present, the cause of MS is not fully understood, but knowledge of the genetic factors involved is essential for effective diagnosis and identification of the most appropriate MS therapeutic interventions. In this study, three genes (*LASP1, TUBA1c*, and *S100A6*) with high degree, high closeness centrality and high betweenness measures were highlighted as potential MS candidate regulator markers. These three genes (*LASP1, TUBA1c*, and *S100A6)* seem to be the most significant in the MS disease process. S100A6 functions in a wide range of cell types as a member of the S100 family and this family expression in MS patients could be considered as a diagnostic biomarker for MS. Its inhibition of demyelinating nerve cells suggests that S100 proteins could act as a candidate therapeutic target in MS (40). Komatsu et al. reported increased expression of S100A6 (Calcyclin), a calcium-bound protein of the S100 family, in human colorectal adenocarcinoma (41). Peterova et al. reported an overexpression of S100 protein-encoding mRNA in both colorectal cancer cell lines and surgically resected specimens of colorectal cancer (42). A study by Bartkowska et al. (43) showed that in response to different stress conditions, the level of *S100A6* decreased in several brain structures, indicating that *S100A6* may modulate stress responses. The genome-wide methylation array has identified a few hypomethylated immune-related genes, amongst them *S100A6* which shows up-regulation in autoimmune encephalitis patients (44). Even though *S100A6* is involved in many biological phenomena, its biological activity is still unknown (45). At the transcriptional level, upstream stimulatory factor and Nuclear factor-kappa B (NF-κB) activates the *S100A6* gene promoter, although p53 might act indirectly to suppress transcription of the *S100A6* gene (46). *TUBA1C* is a member of Microtubules which are vulnerable to degradation and disorganization in a variety of neurodegenerative diseases (47–49). Malfunction of microtubules (e.g., *TUBA1C*) is also considered as the central physiopathological mechanism of neurodegenerative diseases. The abnormalities in the regulatory pathways of microtubules disrupt the properties and functions of microtubules, leading to nerve damage (50). A decreased expression of the *TUBA1C* gene in Parkinson's disease has already been demonstrated by quantitative analysis of gene expression (51). *LASP1*)*The LIM and SH3 protein 1*), a focal adhesion adaptor protein, is an actin-binding, signaling pathway-regulated phosphoprotein which localizes within multiple sites of dynamic actin assembly. It has the potential to interact with various molecules, and is highly expressed in the adult CNS. Microarray data has revealed that alterations in LASP1 proteins affect cell migration, adhesion, and cytoskeletal organization (52). *LASP1*, significantly expressed by CNS neurons, is localized at synaptic sites (53).

A couple of significant transcription factors (TFs) that interact with these hub genes were identified in this study. The YY1 TF (Yi and Yang 1) is a multifactorial protein that, depending on the cell tissue, can activate or suppress gene expression (54). It is expressed in the nervous system. The YY1 promoter lacks the usual TATA box but has a rich GC sequence and therefore resembles a large subset of housekeeping and growth regulator genes. These features suggest that it may play an important role in development. In the CNS, myelination is performed by oligodendrocytes. YY1 function in oligodendrocytes was first reported by Berndt et al. (55). YY1 activates the promoter of myelin lipids and has been identified as an important player in myelination of the central nervous system during growth. In multiple neurodegenerative diseases, YY1 function is degraded through distinct mechanisms, including protein utilization, protein degradation, and ectopic nuclear/cytoplasmic shuttle (N/C). These disorders inhibit YY1 transcriptional activity and lead to gene transcriptional abnormalities that contribute to disease pathogenesis. A future goal in YY1 research is to discover other potential mechanisms that lead to YY1 dysfunction in neurodegenerative diseases, such as ectopic changes after translation (56). The other TF identified in study was Nuclear Factor of Activated T Cells 3 (NFATc3), a member of NFAT family. NFATc3 acts as signal integrators because their function is to bind STAT3, c-Jun, CREB, and ATF3 factors at specific DNA binding sites. NFATc3 cannot be regulated alone and act as calcium-dependent transcription factors. The antigen-mediated T cell receptor (TCR) mediates multiple signaling cascades, including phospholipase C (PLC) -dependent pathways that are secondary messengers of inositol-1,4,5-triphosphate (IP3) and diacylglycerol (DAG). IP3 binds to the IP3 receptor in the endoplasmic reticulum (ER) and releases Ca^2+^ ions into the cytoplasm (57). In this way, NFATc1-4, activates intracellular calcium *via* dephosphorylation (35). The findings show that NFATc3 is defined as a marker of a specific subset of astrocytes that are activated in response to lesions, as well as some degree of heterogeneity among astrocytes that may have consequences for cells in the nervous system (58). Preliminary findings in neuroblast cells have shown that various treatments that alter tubulin polymerization, such as reducing the mineral zinc, prevent the transfer of NFATc3 to the nucleus. In agreement with a functional relationship between NFAT and microtubules, it has been observed that the degradation of several proteins that control the proper organization of the microtubule network, and the actin-cytoskeletal linker, disrupts the nucleus and transcriptional activity of NFAT. Overall, it indicates the involvement of microtubules in NFAT nuclear stimulation (59). The *LASP1* gene enhances NFAT2 nuclear translocation by activating the nuclear factor Akt (60). NFAT can affect processes such as axon growth, synaptogenesis, Schwann cell differentiation, and myelination (58). In general, it can be concluded that increase of the expression of *LASP1* and *S100A6* genes and decrease the expression of the *TUBA1C* gene in multiple sclerosis disrupts NFAT transcriptional activity. Although the role of NFAT in regulating the immune system is well established, our knowledge of NFAT in human disease is limited. The function of NFAT in other aspects of human immune or inflammatory diseases is also largely unknown (61).

The involvement of hub genes identified in this study in other disorders have been reported as well. Patients with MS are known to suffer from a number of digestive problems (62) and studies have shown that *LASP1* (63) and *S100A6* genes have high expression in the digestive system. A link can therefore be established between the expression of these genes, MS, gastrointestinal problems and possibly other types of human cancers (64). Also, *LASP1* plays a crucial role in the growth and metastasis of gastric cancer and other cancers (52, 63, 65–68). For example, *LASP1* can cause the progression and metastasis of colorectal cancer (CRC), but its mechanism is still unclear (69). A connection between *LASP1* and *S100A* has reported underpinning *LASP1* binds to the calcium-binding protein family (*S100A*) and increases its expression in colon cancer (Kappa = 0.347, *P* < 0.01) (70). On this basis, the present study confirmed the importance of three gene expression patterns (*LASP1, S100A6*, and *TUBA1C*) for understanding the transcriptome complexity of MS. This leads us to conclude that upregulation of *LASP1* and *S100A6* genes along with down-regulation of *TUBA1C* is central to MS pathology. To our knowledge, this is the first report to evaluate the level of expression of the above genes for discovery of a transcriptomic signature for MS disease. These findings provide a potential mechanism for some significant biomarkers responsible for the pathogenesis of MS. However, we still have a long way to go to understand the larger transcriptomic profile for this disease. This study provides initial data to further investigate the possible role of these genes in the pathogenesis of MS.

## 5. Conclusions

Results of the present study indicate that the analysis of gene expression data based on gene-gene interaction networks can provide opportunities to determine the genes involved in MS. The importance of three candidate marker genes in this disease were highlighted. These candidate marker genes, *LASP1, TUBA1C*, and *S100A6*, identified by the biological systems approach, have been further confirmed in the laboratory. The significant difference in the expression of these three genes in patients with MS will help further research on this disease and its treatment. This useful tool can serve as a good starting point for identifying new therapies and understanding the basic mechanisms controlling normal cellular processes and disease pathologies. It is crucial to point out here that for learning Bayesian gene network in this study, we did not separate sets of possible signaling protein molecules and transactional factors beforehand in our data, and consider them to be parents (causatives) in the learned network. By doing so, the learned Bayesian gene network probably would be biologically much more appealing. We aim to do this in a due course in the future.

## Data availability statement

The data presented in the study are deposited in the https://www.ncbi.nlm.nih.gov/geo/query/acc.cgi?acc=GSE17048 repository, accession number GSE17048.

## Ethics statement

All procedures were approved and carried out in accordance with Medical Research Ethics Committee in Iran under code IR.UI.REC.1399.076. The patients/participants provided their written informed consent to participate in this study.

## Author contributions

NK and MG-Z developed the theoretical formalism, designed the model and the computational framework, and analyzed the data. NK performed experimental lab the analytic calculations. MM-B conducted the backbone of the experiment and contributed to the final version of the manuscript. All authors have read and agreed to the published version of the manuscript.

## Footnotes

^1^https://www.ncbi.nlm.nih.gov/geo/.

^2^https://www.r-project.org/ (2022).

^3^https://www.bioconductor.org/ (2003–2022).

^4^https://cytoscape.org/ National Institute of General Medical Sciences (NIGMS).
