# Supplementary figures and images for: Gene network reveals LASP1, TUBA1C, and S100A6 are likely playing regulatory roles in multiple sclerosis
Source: Front Neurol. 2023 Mar 9;14:1090631. doi: 10.3389/fneur.2023.1090631 (PMC10035600; doi:10.3389/fneur.2023.1090631)

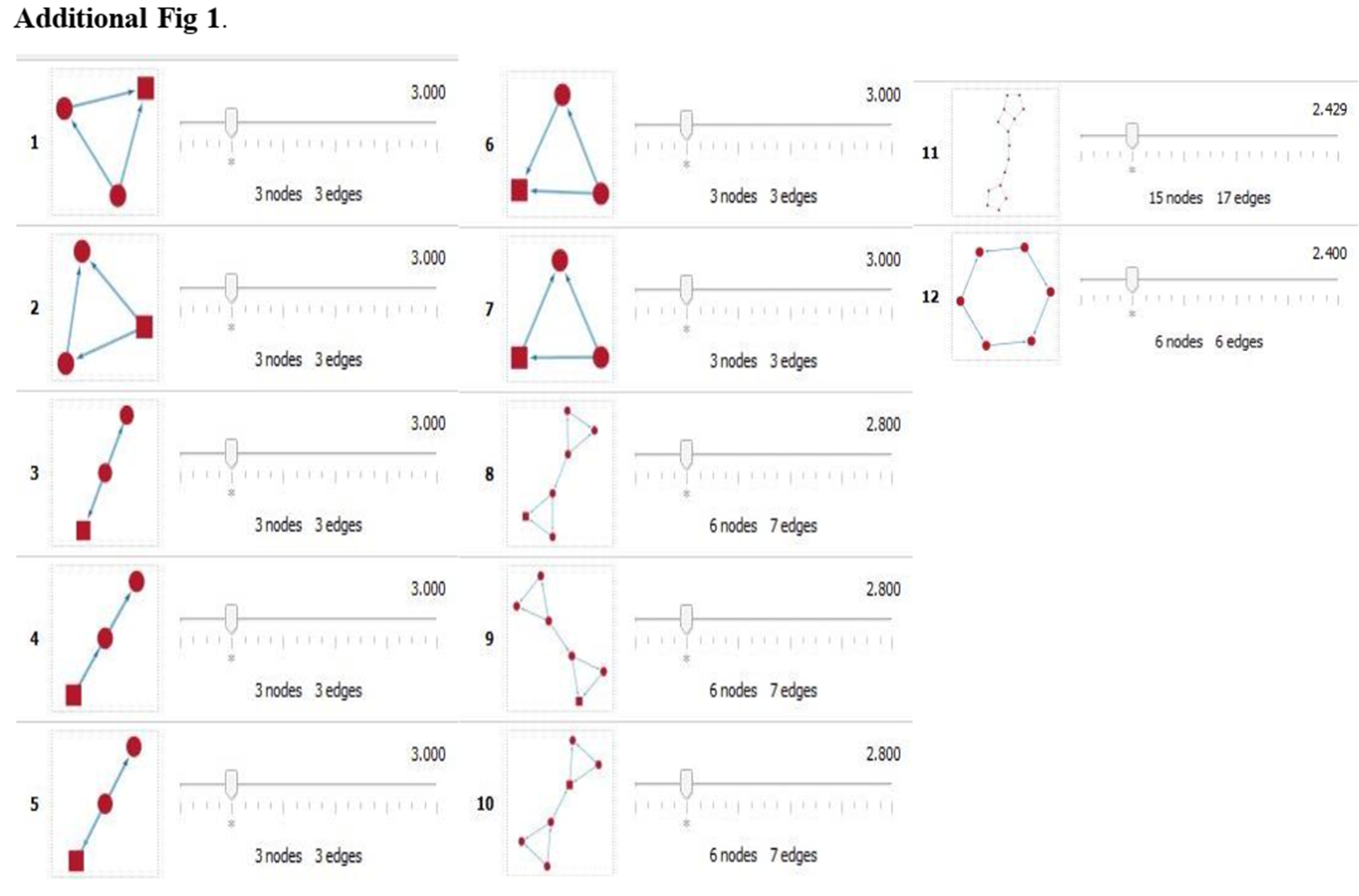

Supplement: Supplementary file 1 [file Image_1.TIF]

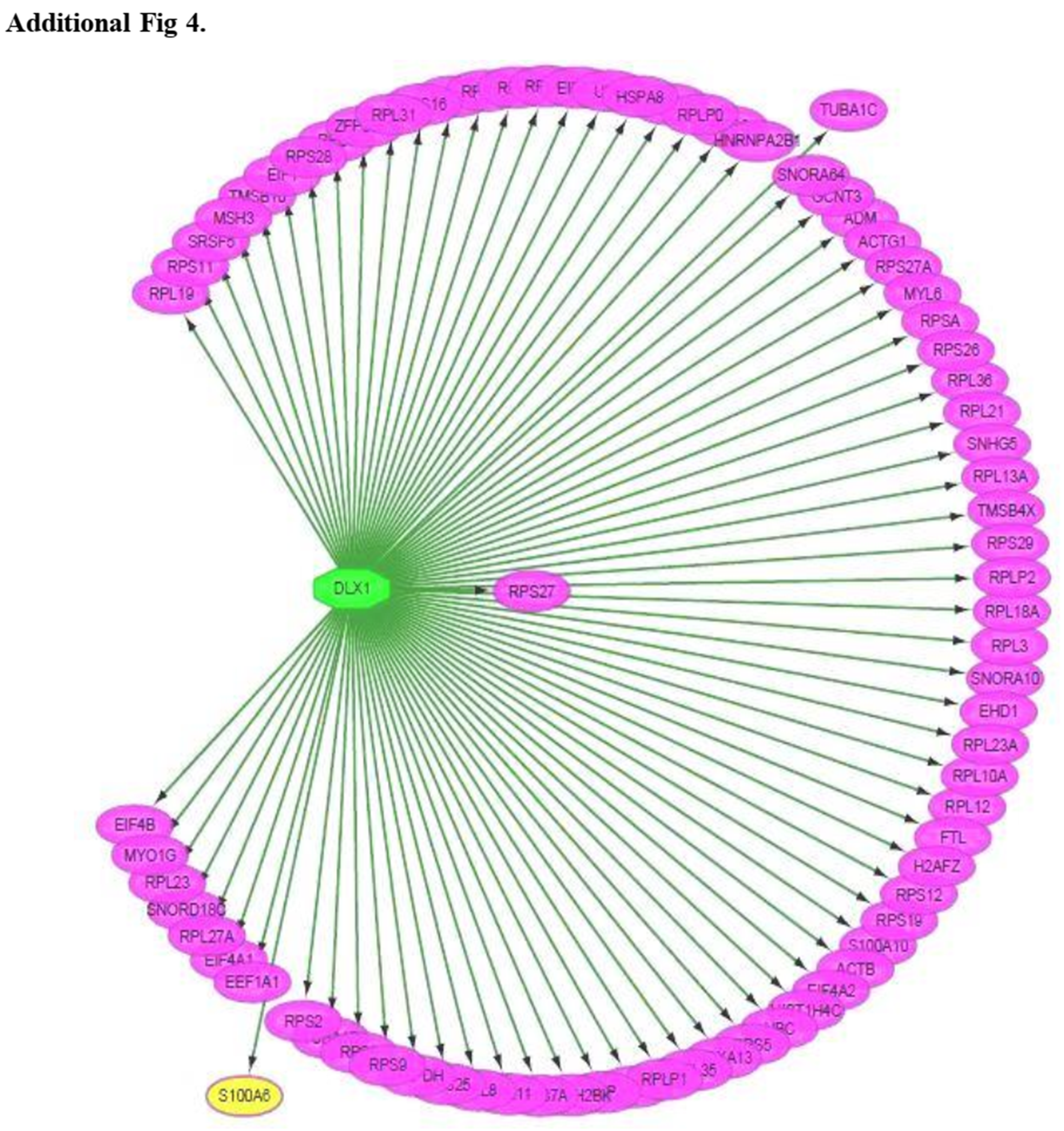

Supplement: Supplementary file 2 [file Image_2.TIF]

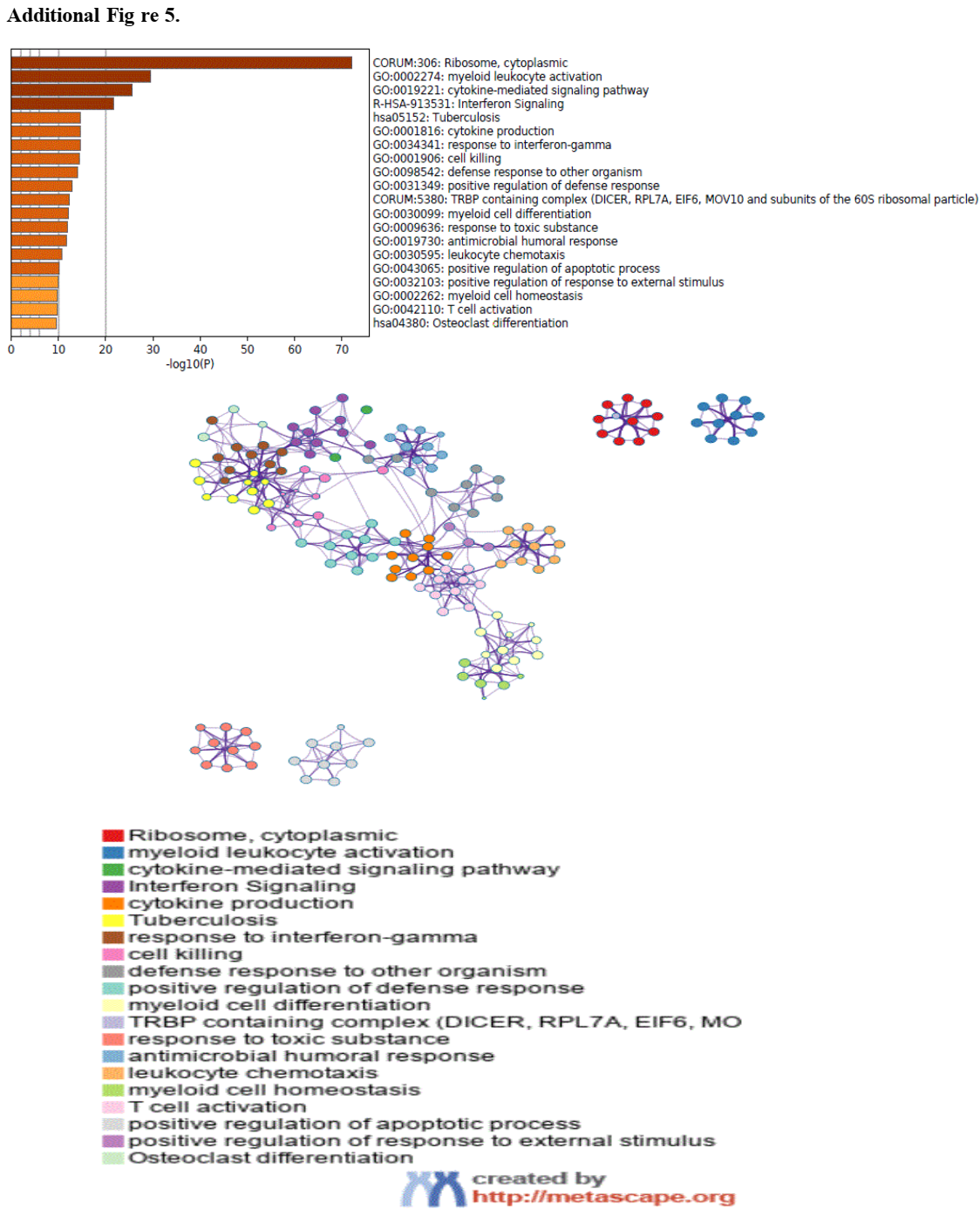

Supplement: Supplementary file 3 [file Image_3.TIF]

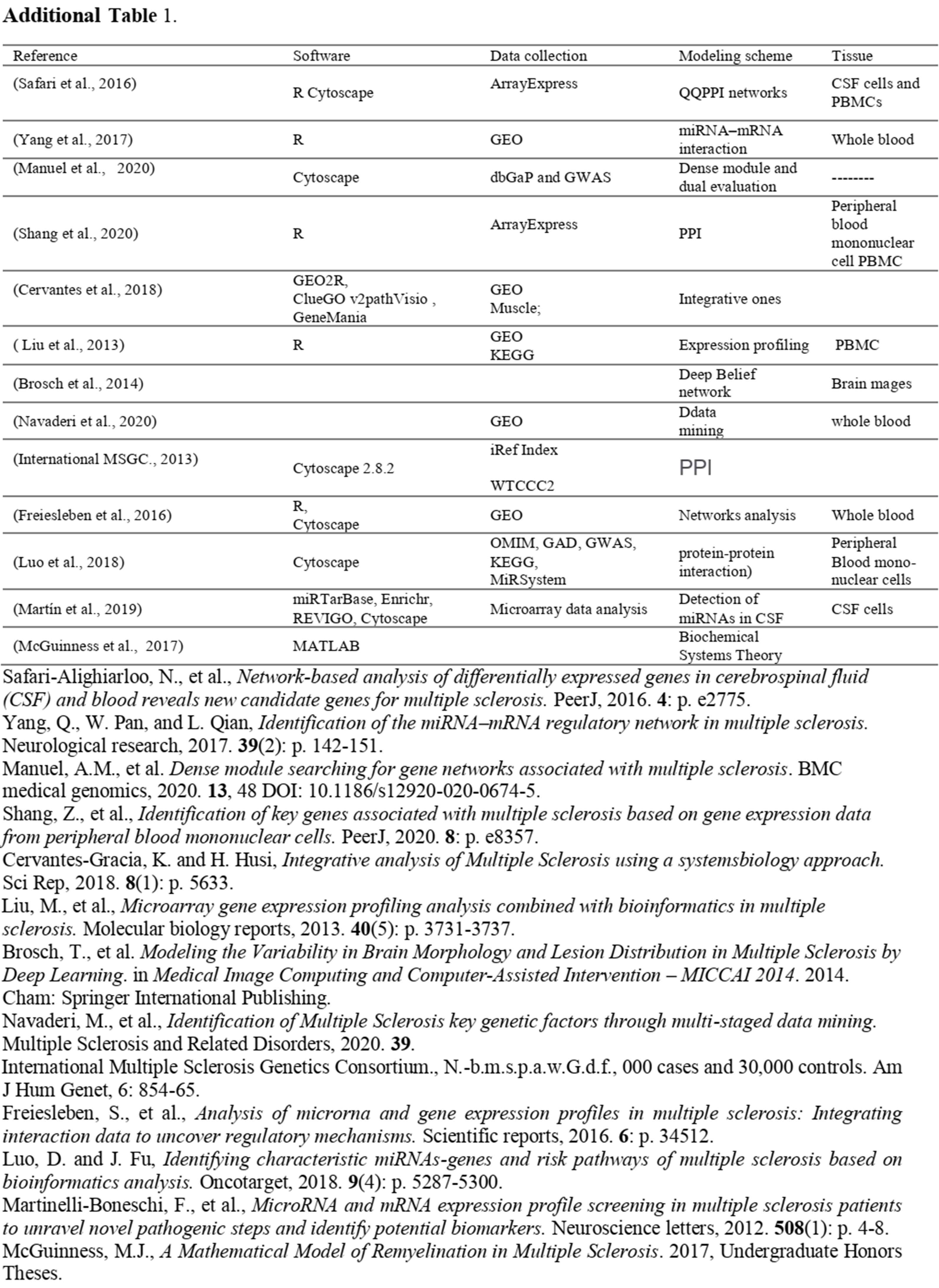

Supplement: Supplementary file 4 [file Image_4.TIF]

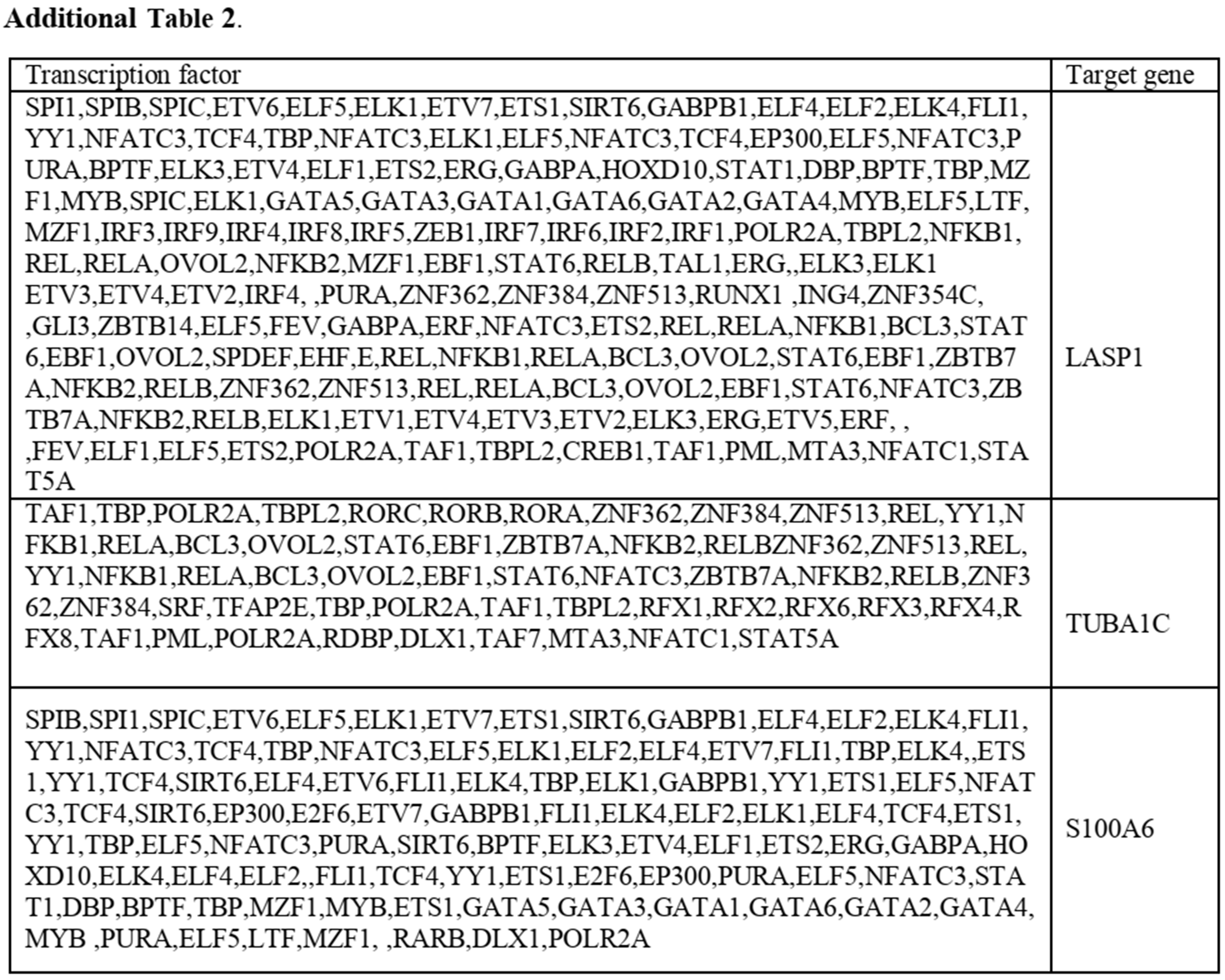

Supplement: Supplementary file 5 [file Image_5.TIF]

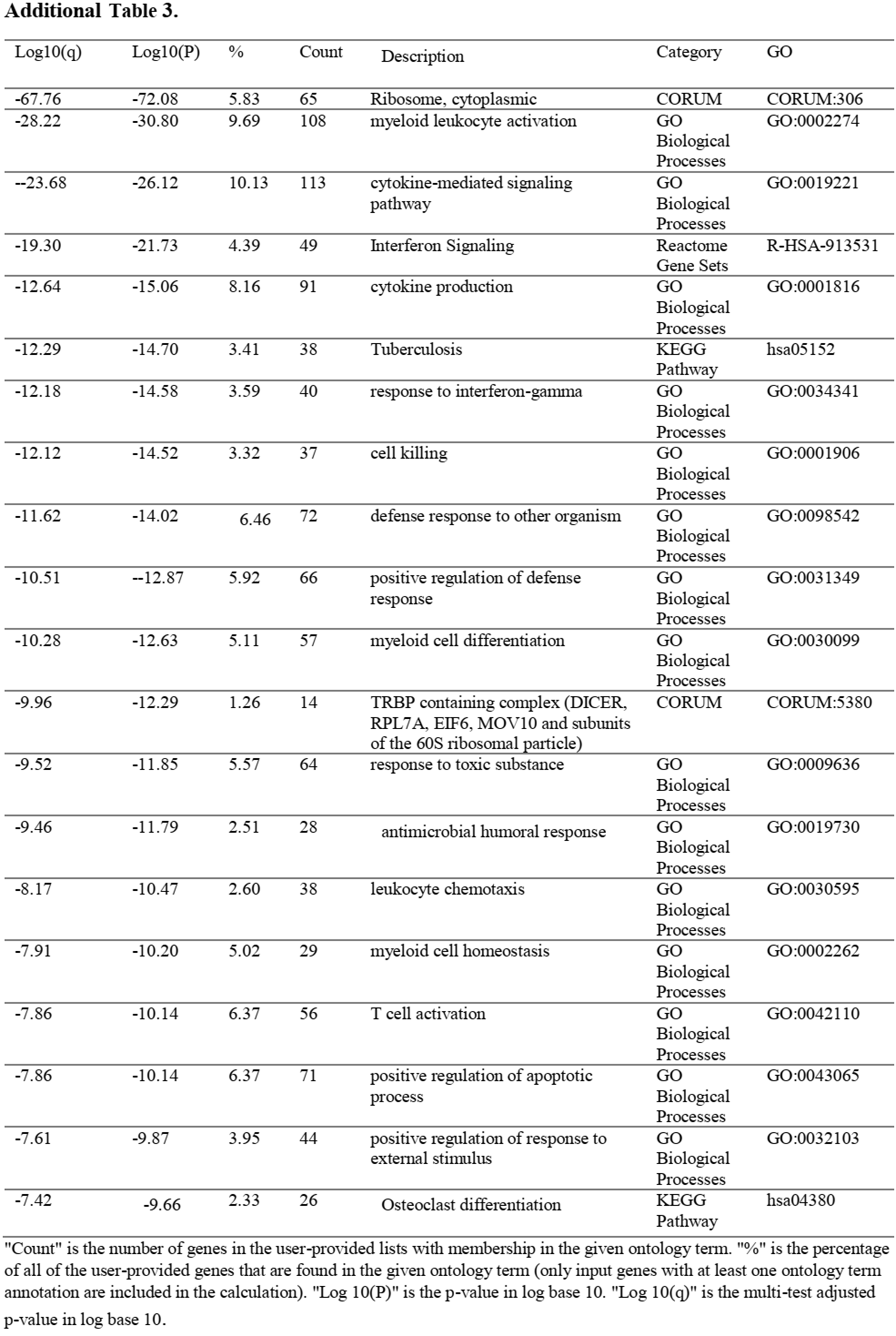

Supplement: Supplementary file 6 [file Image_6.TIF]

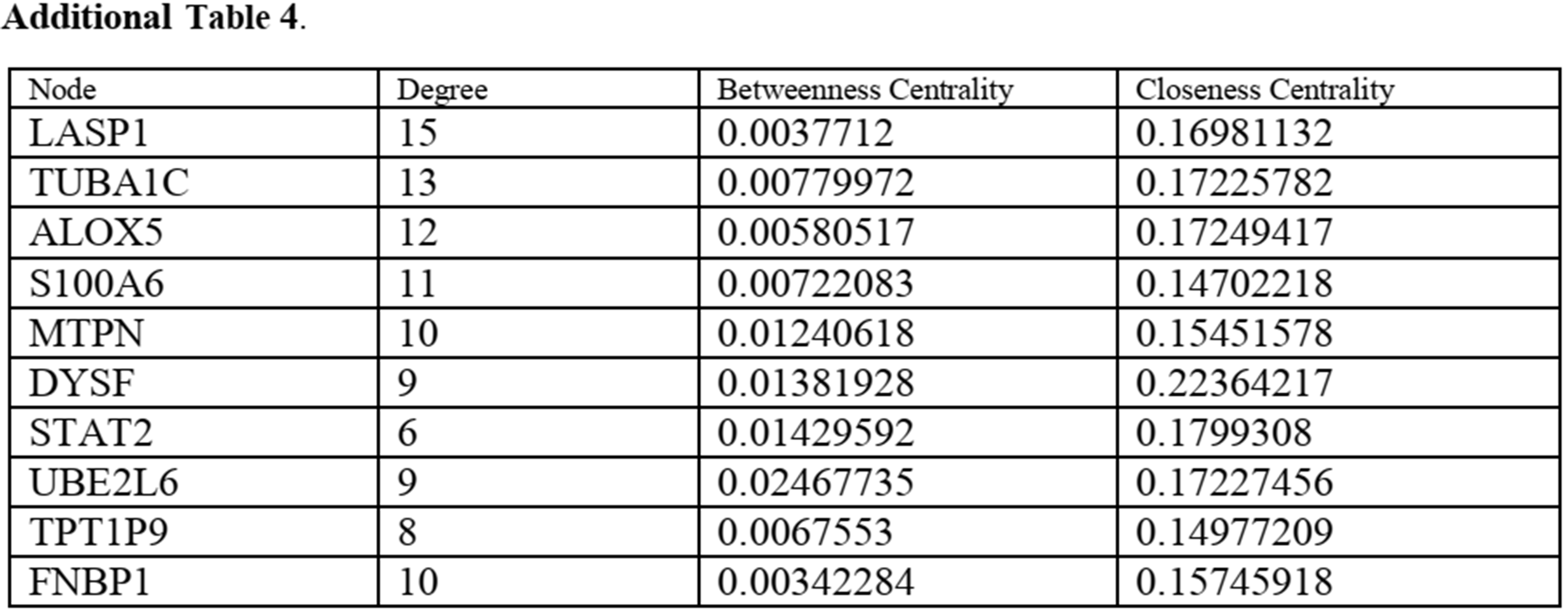

Supplement: Supplementary file 7 [file Image_7.TIF]
